# Design and Measurement of a Novel Overmoded TE_01_ Mode Converter for a Rectangular Gyro-TWT

**DOI:** 10.3390/mi13071111

**Published:** 2022-07-15

**Authors:** Chaoxuan Lu, Wei Jiang, Zewei Wu, Guo Liu, Jianxun Wang, Youlei Pu, Yong Luo

**Affiliations:** School of Electronic Science and Engineering, University of Electronic Science and Technology of China, Chengdu 610054, China; chaoxuanlu@126.com (C.L.); wzw.198704@163.com (Z.W.); liuguo@uestc.edu.cn (G.L.); jxunwang@uestc.edu.cn (J.W.); puyoulei@uestc.edu.cn (Y.P.); yluo@uestc.edu.cn (Y.L.)

**Keywords:** gyro-TWT, rectangular overmoded TE_01_ mode, mode converter

## Abstract

The rectangular gyrotron traveling wave tube (gyro-TWT) with a large aspect ratio (*α*) has the potential to achieve megawatt-class output power. As an essential component of gyro-TWT, a novel overmoded Ka-band mode converter with an *α* of 6.19 is designed, analyzed, and cold tested in this paper. Based on the magnetic dipole moment theory, the rectangular overmoded TE_01_ mode is excited via the rectangular fundamental TE_10_ mode. The cutoff waveguide is applied to prevent electromagnetic wave transport to the magnetron injection gun (MIG) region and also guarantee higher power electron beam transportation. Simulations predict an operation bandwidth higher than 4 GHz and greater than 99.8% mode purity between 33–37 GHz. To verify this design, the mode converter is manufactured and cold tested. The back-to-back measurement results exhibit a good agreement with the simulation. With similar topologies, the rectangular overmoded TE_01_ mode can be excited in a different *α*.

## 1. Introduction

The gyro-TWT as a millimeter-wave source has the characteristics of high power, high frequency, and wide bandwidth. It has promising prospects in high-resolution imaging radar, electronic countermeasures, and subsequent generations of communication systems beyond 5G/6G [1,2,3,4,5].

To improve the performance of the gyro-TWT, many different beam-wave interaction circuits have been designed and reported by several organizations in recent years. Examples include a helically corrugated waveguide [6], confocal waveguide [7], photonic bandgap [8], and dielectric-loaded circuit [9,10,11,12,13,14]. The dielectric-loaded circuit is one of the successful structures that has the possibility to achieve high power output. At the University of Electronic Science and Technology of China (UESTC), we have performed some investigations on the circular dielectric-loaded gyro-TWT [15,16,17,18]. The hot experimental results show that the TE_01_ mode (the sixth mode) Ka-band gyro-TWT, driven by a 60 kV−10 A electron beam, can obtain 150 kW peak power, 10 kW average power, and a 1 dB fraction bandwidth of 8%. However, the maximum output power of these gyro-TWTs is much less than the megawatt class.

As the gyro-TWT develops towards higher output power, especially the megawatt-class output power, problems such as the high beam current density and thermal power capacity on the dielectric-loaded ceramic should be considered and addressed. (1) The radius of the circular interaction circuit is determined by the operating frequency, and the channel area that allows the electron beam to be transmitted is limited in the waveguide. One way to increase the waveguide area is to select the high-order operating mode, like the megawatt gyrotron oscillator (gyrotron) [19,20]. However, the high-order mode can easily cause severe oscillation, making the gyro-TWT unstable [21]. (2) The temperature of the attenuated ceramic will restrict the increase of the output power of the circular gyro-TWT. The heat dissipation on the ceramic is a severe problem due to the limited cooling area when the tube operates at high power [22].

**Figure 1:**
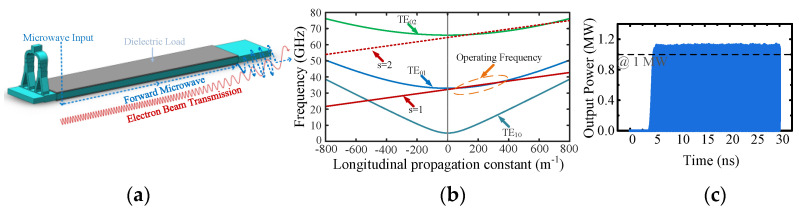
(**a**) Schematic of mode converter and rectangular dielectric-loaded interaction circuit. (**b**) Beam-wave dispersion relations in rectangular gyro-TWT. (**c**) Output power as a function of time.

The investigation of rectangular dielectric-loaded gyro-TWT, as shown in Figure 1a, provides an idea to solve these problems. (1) When the operating mode is rectangular TE_0m_ (m = 1, 2, …) mode, the operating frequency is only determined by the narrow side (*N*). Figure 1b shows the dispersion diagram of the TE_01_ mode rectangular gyro-TWT operating between 33–37 GHz. Under a higher beam current, the channel area allowing beam transport can be enlarged, and the beam current density can be reduced effectively by enlarging the wide side (*W*). (2) When the *W* and *N* are 30 and 4.85 mm, respectively, the operating mode order is not higher (the seventh mode), the corresponding mode density is low, and the mode competition can be suppressed effectively. With a 60 kV−48 A electron beam, the rectangular gyro-TWT can obtain more than 1 MW output power in the Particle-in-Cell (PIC) simulation, as shown in Figure 1c. (3) Compared with the circular gyro-TWT in [16], the current is 4.8 times higher than before, but the corresponding current density is almost unchanged due to the increased waveguide area. (4) When the gyro-TWT operates at high power, the heat on the rectangular ceramic can also be efficiently taken away by the external coolant due to the large heat dissipation area.

As shown in Figure 1a, when the gyro-TWT is operating, the electron beam is emitted from the MIG, which then interacts with the microwaves (input from the mode converter) in the dielectric-loaded circuit. Finally, the signal is amplified and output, and the electron beam is collected. The mode converter is an essential component of the gyro-TWT, and its performance significantly influences the tube. It is generally required to have the characteristics of high mode purity, wide bandwidth (larger than the operating bandwidth of the gyro-TWT), and high converting efficiency. There are various mode converter designs in the gyro-TWT. With the T-junction structure, a TE_11_ mode side coupler with 10.5% relative bandwidth is designed for a W-band gyro-TWT [23]. A T-junction input coupler with Bragg reflector and a multiple-hole directional coupler for a low-terahertz gyro-TWT achieve relative bandwidths of 8% and 28% [24]. Some high-order waveguide modes such as TE_01_, TE_21_, TE_02_, TE_13,_ and TE_41_ can be launched by the Y-type structure [25,26,27,28]. By the coupling apertures, the TE_01_ and TE_13_ mode converters are designed and measured at Q-band and W-band. The relative bandwidth of the two mode converters is 11.3% and 10.3%. The circular TE_01_ mode is achieved via the rectangular TE_20,_ and, with a similar topology, the circular TE_13_ mode is achieved via the rectangular TE_50_ mode [29]. Some other mode conversion methodologies for the gyro-TWT have also been reported, such as circular TM_01_ mode excitation based on a ridge gap waveguide [30], and HE_11_ hybrid mode excitation based on scattering matrix formalism for high-power radar applications [31]. The performance comparison of some proposed mode converters for gyro-TWT is listed in Table 1.

However, there are currently few reports on large α overmoded mode converters for rectangular dielectric-loaded gyro-TWT. For the study and application of Ka-band megawatt-class gyro-TWT, a novel rectangular overmoded TE_01_ mode converter based on the magnetic dipole moment theory is investigated in this paper. This large size mode converter (*W* = 30 mm, *N* = 4.85 mm) can launch the overmoded TE_01_ mode by using similar topologies. In addition, the TE_n0_ (*n* = 1, 2, 3, …) modes are also suppressed effectively. The application of the cutoff waveguide with a simple structure can prevent microwave transmission to the MIG region, while the transportation of a high-power electron beam is also satisfied. The details of theoretical analysis, design, and simulation are described in Section 2. Section 3 shows the back-to-back measurements and the comparison of the results between the simulation and cold test. The final section comprises a conclusion.

## 2. Overmoded TE_01_ Excitation

### 2.1. Design Principle

**Figure 2:**
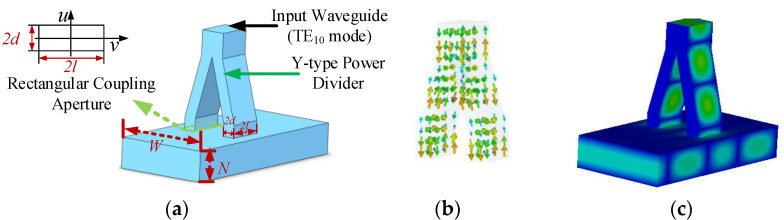
Schematic of a TE_01_ mode converter. (**a**) Vacuum structure. (**b**) Magnetic field distributions in the power divider. (**c**) Electric field distribution in the mode converter.

To effectively obtain the overmoded rectangular TE_01_ mode converter with large *α*, the principle demonstration of the mode excitation is carried out for a small *α* mode converter. As shown in Figure 2, it is the schematic of the mode converter, and the magnetic dipole moment theory is adopted. The mode converter is mainly composed of a Y-type power divider, two rectangular coupling apertures, and an overmoded output waveguide. The two coupling apertures are symmetrical to the center of the E-plane of the overmoded output waveguide. The dimensions of the input waveguide (TE_10_ mode input) are determined by the coupling aperture: the narrow side of the input waveguide is twice that of the coupling aperture, and the wide side is the same as the coupling aperture.

When the aperture is smaller than the wavelength of the electromagnetic wave, the coupling aperture can be equivalent to a combination of the electric and magnetic dipole. The electric dipole moment (*P*) and magnetic dipole moment (*M*) are proportional to the normal electric field (*E*_1*n*_) and tangential magnetic field (*H*_1*u,v*_) of the incident wave, respectively. The mode of the incident wave in the main waveguide is represented by subscript 1, and the mode excited in the secondary waveguide is represented by subscript 2 [28,30,32,33,34].

(1) $$P=-\varepsilon_{0}p_{n}E_{1n}$$

(2) $$M=m_{u}H_{1u}+m_{v}H_{1v}$$

Here *ε*_0_ is the vacuum permittivity, and *p_n_* is normal polarizability. *m_u_* and *m_v_* are orthogonal tangential magnetic polarizabilities, which depend on the shape and size of the coupling apertures. The corresponding coupling (*β*) can be expressed as:

(3) $$\beta =-j\frac{\omega }{2}(\mu_{0}m_{u}H_{1u}H_{2u}+\mu_{0}m_{v}H_{1v}H_{2v}+\varepsilon_{0}p_{n}E_{1n}E_{2n})$$

Here *μ*_0_ is the vacuum permeability, and *ω* is the angular frequency of the wave. *E*_2*n*_ and *H*_2*u,v*_ are the normal electric and tangential magnetic fields of the excited mode, respectively. For the rectangular coupling aperture:

(4) $$\left\{ \begin{array}{l} m_{u}=0\\ m_{v}=\frac{\pi }{2}ld^{2}\\ p_{n}=\frac{\pi }{2}ld^{2} \end{array}\right.$$

*l* and *d* are half of the wide and narrow dimensions of the rectangular coupling aperture. According to the coupling characteristics of the apertures designed in this paper, *E*_1*n*_ is zero, and *H*_1*t*_ is nonzero. Therefore, the coupling can be expressed as:

(5) $$\beta =-j\frac{\omega }{2}\mu_{0}m_{v}H_{1v}H_{2v}$$

The electric field direction of the TE_10_ mode in the coupling aperture is parallel to the TE_01_ mode of the secondary waveguide. For the TE_n0_ mode in the secondary waveguide, the electric field is perpendicular to the E-plane. With properly arranged magnetic dipoles in the rectangular waveguide, the coupling structure mentioned can prevent the TE_n0_ modes excitation, and the TE_01_ mode would be generated.

### 2.2. Simulation and Analysis

**Figure 3:**
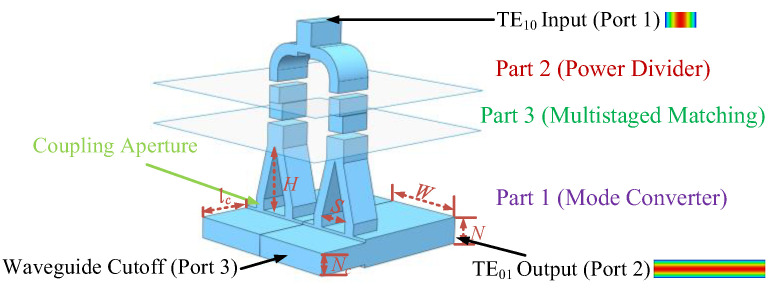
Schematic of a TE_01_ mode converter for rectangular gyro-TWT.

In order to obtain an overmoded TE_01_ mode converter with good performance conveniently and efficiently, we divide the design process into three parts, as shown in Figure 3. Part (1) consists of designing a small *α* mode converter and combining the ones with similar topologies to achieve a large *α* (*N* unchanged) for rectangular gyro-TWT operation. Part (2) is the design and simulation of a power divider. The input port of the component is the standard Ka-band waveguide, which connects the external solid-state power amplifier. Part (3) optimizes the multistaged matching. The input port size of the mode converter may be different from the output port of the power divider, so the multistaged matching is designed to connect the mode converter and power divider.

The dimensions of the mode converter are determined by the interaction circuit of the rectangular gyro-TWT (*W* = 30 mm, *N* = 4.85 mm, *α* = 6.19). In this paper, the mode converter for Ka-band gyro-TWT operation can be achieved by combining two small *α* overmoded converters (*W* = 15 mm, *N* = 4.85 mm). The Y-type power divider is chosen to ensure that the two signals (TE_10_ mode) delivered to the mode converter have equal amplitude and the same phase. The rectangular TE_10_ mode is input from the standard Ka-band waveguide and divided into four signals by the Y-type power dividers. The four signals will couple into the interaction circuit and excite the operating mode.

As shown in Figure 1a, for the rectangular gyro-TWT with a high beam current, the input microwave propagates into the MIG region and may cause the gyro-TWT to be unstable. The shorter the distance the microwaves travel in the MIG region, the lower the possibility that the gyro-TWT will oscillate. The cutoff waveguide as a simple structure is easy to fabricate and can reflect the TE_01_ mode to the interaction circuit. According to the operating bandwidth of the gyro-TWT (33–37 GHz), the narrow side of the cutoff waveguide (*N_c_*) is designed to be 3.8 mm, and the corresponding cutoff frequency is about 39.5 GHz. The length of the cutoff waveguide (*l_c_*) is 10 mm and lower than the starting length. The area of the cutoff waveguide can guarantee a higher power electron beam transportation and suppression for the operating mode.

**Figure 4:**
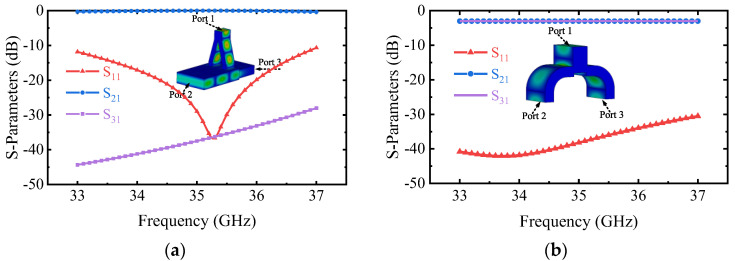
Simulated S-parameters of the (**a**) Small *α* mode converter. (**b**) Y-type power divider.

Based on the design principle, the magnetic dipole arrangement of TE_01_ mode will be obtained by designing the critical dimensions *d*, *l*, and spacing of coupling apertures (*S*) in the small *α* mode converter. Taking the transmission coefficient of the desired mode S_21_ as the goal (S_21_ is as high as possible), these parameters are optimized by the multi-objective genetic algorithm (MOGA) of the commercial software CST-Microwave Studio (CST-MWS) [35], and the final *d*, *l*, *s* are 1.005 mm, 2.9 mm, and 5.48 mm, respectively. The simulation results are shown in Figure 4a. S_21_ of the operating mode is more than −0.4 dB, and the port reflection S_11_ is lower than −10.5 dB between 33–37 GHz. The mode purity exceeds 99.8%. The reflection of port 1 reaches up to −36.8 dB at 35.3 GHz. In addition, the operating mode to port 3 is lower than −25 dB, which effectively suppresses the transmission of the electromagnetic wave to the MIG region. S_21_ of other modes is lower than −30 dB. The mode converter with any *α* can be achieved by combining similar small *α* mode converters together.

After determining the position of the mode converter input port, a Y-type power divider is designed and simulated. As shown in Figure 4b, the S_21_ is greater than −0.45 dB, and the S_11_ is lower than −30 dB between 33–37 GHz. The results indicate that the performance of this component does not affect the overall performance of the mode converter.

**Figure 5:**
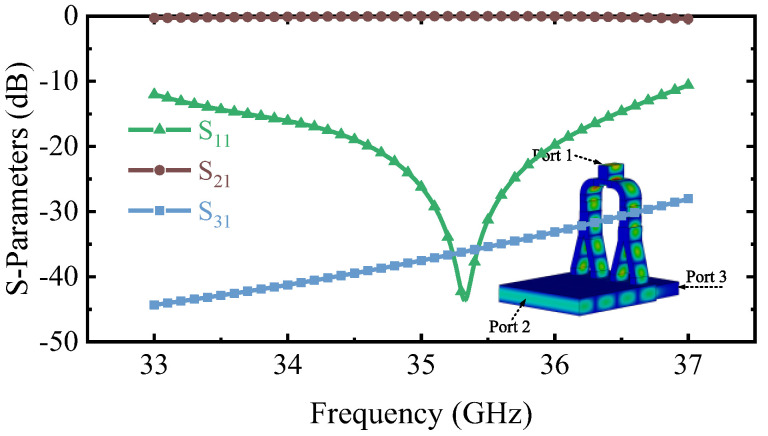
Simulation results of the mode converter for rectangular gyro-TWT operation.

The multistaged matching is optimized to reduce the effects of port mismatches between the power divider and mode converter. The two parts are connected by multistaged matching, and the simulation results of the mode converter with large *α* are shown in Figure 5. S_21_ of the overmoded TE_01_ mode is higher than −0.45 dB, and S_11_ is lower than −10.5 dB between 33–37 GHz. Simulations predict an operation bandwidth higher than 4 GHz. The mode purity exceeds 99.8%, and the mode conversion efficiency is greater than 95%. The S_11_ reaches up to −43.6 dB at 35.3 GHz, and the S_11_ is up to −0.011 dB. The results indicate that the overmoded mode converter has a good performance. Moreover, the simulation results are also similar to the small *α* mode converter and demonstrate the feasibility of the method to obtain the mode converter with a different *α* by using similar topologies.

## 3. Manufacture and Microwave Measurement

**Figure 6:**
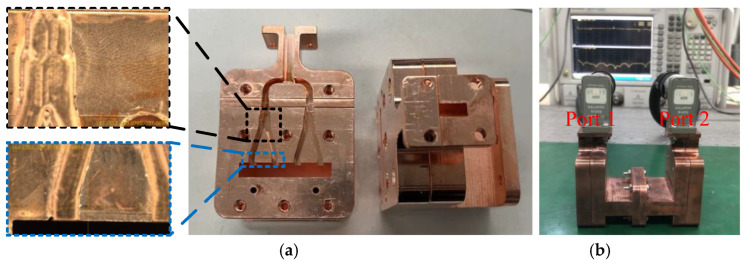
(**a**) Manufacture and partially enlarged view of the Y-type power divider and rectangular aperture. (**b**) Measurement of the mode converter.

In Figure 6a, two identical Ka-band overmoded TE_01_ mode converters made of oxygen-free high-conductivity (OFHC) copper block are fabricated. To make the simulation model more consistent with the actual assembled model, the specific dimensions of a mode converter are also measured with an optical microscope. The dimension comparison between the simulation and assembled model is shown in Table 2. The observation results indicate that the actual size does not deviate much from the design, but parts of the Y-type power dividers and rectangular coupling apertures have large machining errors or even damage. The measured key dimensions of the mode converter are brought into the simulation model.

The surface roughness of the mode converter will affect the transmission coefficient of the desired mode. Based on the Hammerstad–Bekkadal (HB) formula analytical model, the effective conductivity (*σ*) is introduced to analyze the conductor loss caused by rough metallic surfaces [36].

(6) $$\sigma_{eff}=\sigma_{D}\cdot {\left\{1+\frac{2}{\pi }\arctan\left[1.4{\left(\frac{h}{\delta }\right)}^{2}\right]\right\}}^{-2}$$

Here, *σ_D_* is the conductivity of a flat surface, *σ_eff_* is the effective conductivity of the rough surface, *δ* is the skin depth in the case of a flat surface, and *h* is the root-mean-square height of the surface. The calculated *σ_eff_* is about 1.54 × 10^7^ S/m when the surface roughness is about 300 nm level.

**Figure 7:**
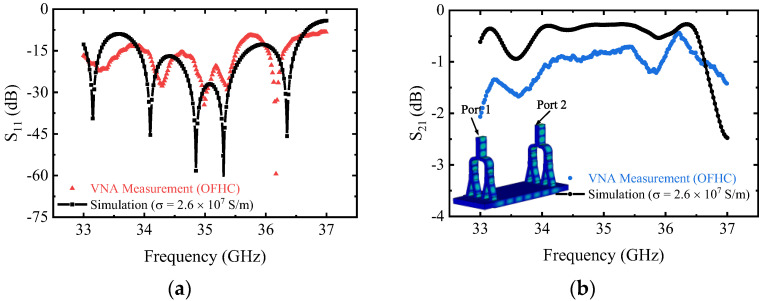
Comparison of the simulation and measurement results of the mode converter (**a**) S_11_ and (**b**) S_21_.

The back-to-back measurement method is adopted to verify the port reflection and transmission coefficient. The two mode converters are connected with the vector network analyzer (VNA) and tested as shown in Figure 6b. Ports 1 and 2 are connected to the VNA (working from 0 to 67 GHz), and the ports of waveguide cutoff are open to the air. The comparison of the simulation and measurement results of the mode converter is shown in Figure 7. It can be seen that the measured and simulated results are in good agreement in the frequency range of 33 to 37 GHz, after considering the surface roughness. The measured port reflection S_11_ is lower than −10 dB except for the high-frequency point, and the frequency is slightly shifted compared with the simulation. The measurement result of transmission coefficient S_21_ is almost greater than −2 dB and is a little worse than the simulation results. The discrepancy between the simulated and measured results is probably caused by some damaged Y-type power dividers and rectangular apertures, as shown in Figure 6a. The leakage of the microwave caused by assembly errors in the test may also be the reason for the poor S_21_.

## 4. Conclusions

In this paper, a novel overmoded rectangular TE_01_ mode converter is analyzed, simulated, and tested. By the design of a small *α* mode converter, power divider, and multistaged matching, the overmoded TE_01_ mode converter (*α* = 6.19) for rectangular gyro-TWT operation is obtained efficiently. The measurement results indicate that S_11_ is almost lower than −10 dB and the S_21_ is almost more than −2 dB between 33 to 37 GHz and these are also in good agreement with the simulation results. The mode conversion with different *α* can also be achieved by combining similar topologies. The investigation of the mode conversion supports the development of the rectangular gyro-TWT.

## Figures and Tables

**Table 1:** Performance comparison of different mode converters.

Structure Type	Mode Conversion	Frequency Band	Relative Bandwidth
T-junction	$TE_{10}^{□1}\to TE_{11}^{○2}$	W-band	10.5% [23]
Y-type	$TE_{10}^{□}\to TE_{13}^{○}$	Q-band	4.4% [26]
Multiple-hole	$TE_{10}^{□}\to TE_{11}^{○}$	Y-band	28% [24]
Coupling aperture	$TE_{10}^{□}\to TE_{01}^{○}$	Q-band	11.3% [29]
This work	$TE_{10}^{□}\to TE_{01}^{□}$	Ka-band	11.4%

^1^ The symbol □ represents the rectangular waveguide. ^2^ The symbol ○ represents the circular waveguide.

**Table 2:** Comparison of some dimensions between the design and actual model.

Parameter	*W*	*N*	*s*	*l*	*d*	*H*
Design (mm)	30	4.85	5.48	2.9	1.005	11.5
Actual (mm)	29.97	4.86	5.48	2.91	1.015	12

## Data Availability

All data included in this study are available upon request by contacting with the corresponding author.
